# Assessment of proteomic salivary biomarkers for diagnosis of early childhood caries: a systematic review

**DOI:** 10.3389/froh.2025.1695189

**Published:** 2026-01-06

**Authors:** P. K. Shivaprakash, Sushmitha Dandamudi, P. Tharani, Anitha R. Sagarkar, Sunaina Shetty Yadadi, Vineet Vinay, Vijay Desai, Tarun Walia, Raghavendra M. Shetty

**Affiliations:** 1 Department of Pediatric and Preventive Dentistry, P.M.N.M Dental College and Hospital, Bagalkot, India; 2 Hospital for Children and Women, Hyderabad, India; 3 Dr. Barun’s Multispecialty Dental Center, Chennai, India; 4 Department of Public Health Dentistry, Faculty of Dental Sciences, MS Ramaiah University of Applied Sciences, Bengaluru, India; 5 Department of Restorative Dentistry, College of Dental Medicine, University of Sharjah, Sharjah, United Arab Emirates; 6 Department of Public Health Dentistry, Sinhgad Dental College and Hospital, Pune, India; 7 Department of Clinical Sciences, College of Dentistry, Ajman University, Ajman, United Arab Emirates; 8 International Adjunct Faculty, Department of Pediatric and Preventive Dentistry, Sharad Pawar Dental College and Hospital, Datta Meghe Institute of Higher Education and Research (Declared as Deemed-to-be University), Wardha, India

**Keywords:** early childhood caries (ECC), severe early childhood caries (s-ECC), salivary biomarkers, proteomics, peptidomics, diagnostic biomarkers, risk prediction

## Abstract

**Background:**

Severe early childhood caries (s-ECC) remains a highly prevalent condition among children worldwide, posing a considerable burden in both developed and developing regions. This systematic review seeks to comprehensively identify, synthesize, and critically evaluate available evidence on salivary proteomic and peptidomic biomarkers that may aid in the diagnosis or risk assessment of ECC.

**Aim and objectives:**

This study aims to evaluate any difference in the level of proteomic salivary biomarkers in children with and without ECC.

**Methods:**

A comprehensive search was conducted across four major electronic databases: the Cochrane Central Register of Controlled Trials (CENTRAL), MEDLINE via PubMed, Scopus, and Google Scholar. From an initial yield of 1,253 records, 11 studies fulfilled the eligibility criteria and were included for data extraction. The risk of bias for the included studies was assessed using the QUADAS-2 tool for the Quality Assessment of Diagnostic Accuracy Studies.

**Results:**

Of the 11 studies included, quality appraisal using the QUADAS-2 indicated that 3 out of 11 studies had low risk in all domains, while the remaining studies had unclear or high-risk domains.

**Conclusion:**

The pooled evidence suggests that salivary biomarkers have significant potential for detecting ECC. Utilizing salivary diagnostics may enhance the prevention of new carious lesions. Future clinical trials with robust methodologies are recommended to generate further conclusive evidence.

**Clinical significance:**

Salivary proteomic biomarkers, including MS protein, mucins, amylase, glycoproteins, proline, and glycine, may provide additional value in the early diagnosis of ECC in pediatric populations.

**Systematic Review Registration:**

https://www.crd.york.ac.uk/PROSPERO/view/CRD42023468566, PROSPERO CRD42023468566.

## 1 Introduction

Dental caries is one of the most common yet largely preventable oral diseases worldwide, making it a major public health concern (1, 2). Conventional restorative approaches for managing tooth decay impose a substantial economic burden on individuals and healthcare systems (3). In cases of severe early childhood caries (s-ECC), treatment frequently requires general anesthesia, which carries additional risks of morbidity and even mortality (4). As dental caries is a multifactorial disease, preventive strategies that rely solely on patient-driven behavioral modifications, such as dietary control and oral hygiene, are often insufficient.

ECC is a complex, infectious condition that remains difficult to manage, with multiple studies highlighting the limitations of existing preventive and therapeutic measures (5). The definition of s-ECC is “(1) any sign of smooth-surface caries in a child younger than 3 years of age; (2) from ages 3 through 5 years, 1 or more cavitated, missing (secondary to caries), or filled smooth surfaces in primary maxillary anterior teeth; or (3) a decayed, missing, or filled score of greater than or equal to 4 (age 3), greater than or equal to 5 (age 4), or greater than or equal to 6 (age 5)” (6). The consequences of s-ECC extend beyond tooth decay, as it can compromise children's ability to eat and drink properly, contribute to malocclusion in the permanent dentition (7, 8), and lead to secondary complications such as malnutrition, sleep and speech difficulties, poor academic performance, social challenges, and reduced self-esteem (9). Collectively, these effects significantly impair the quality of life of preschool children and their families (8, 10).

Saliva, despite being one of the body's key protective fluids, remains relatively underexplored in caries research. Earlier investigations primarily examined the physiochemical properties of saliva (e.g., flow rate and buffering capacity) or specific antimicrobial constituents such as immunoglobulin A, lactoferrin, lysozyme, and the peroxidase–hypothiocyanate system, in relation to caries susceptibility in children (11).

The quantitative assessment of compounds containing amine functional groups plays a crucial role in diagnostics (12). Within dentistry, identifying these biomolecules may provide deeper insights into the functional mechanisms of oral microorganisms (13–15) and their ability to persist in a complex and dynamic environment. Previous research has reported the presence of free amino acids in saliva (16, 17), with several studies attempting to associate these findings with caries experience (18).

In the era of evidence-based dentistry, establishing robust evidence on combined salivary proteomic and peptidomic biomarkers for the diagnosis or risk assessment of ECC is critically needed. Investigating biomarkers that can detect cariogenic microbes and related proteins is essential and requires a comprehensive study. Therefore, the present systematic review aimed to evaluate differences in salivary proteomic biomarker levels between children with and without ECC, while systematically identifying, synthesizing, and critically appraising studies that have examined salivary proteomic and peptidomic biomarkers for ECC diagnosis or risk prediction.

## 2 Materials and methods

The present systematic review was reported in accordance with the updated Preferred Reporting Items for Systematic Reviews and Meta-Analyses (PRISMA) guidelines. The protocol of the current review was registered in PROSPERO (CRD42023468566).

### 2.1 Search strategy and study selection

A comprehensive systematic search was conducted following PRISMA guidelines to identify relevant studies investigating salivary proteomic biomarkers in ECC. The search encompassed three major electronic databases: Cochrane Central Register of Controlled Trials (CENTRAL), MEDLINE via PubMed, Scopus, and Google Scholar. Other relevant sources, such as gray literature, were explored. The search strategy employed Medical Subject Headings (MeSH) terms combined with free-text keywords and Boolean operators (AND, OR). Key search terms included the following: “early childhood caries” OR “ECC” OR “dental caries in children” AND “salivary biomarkers” OR “proteomics” OR “salivary proteins” AND “diagnosis” OR “detection” OR “risk assessment.” The search was restricted to English-language articles published up to 30 May 2025, to ensure inclusion of contemporary evidence while maintaining linguistic consistency. No geographic restrictions were applied to maximize global representation of studies.

For PubMed, the following search strategy was used: (((“Early Childhood Caries”[Mesh] OR “Dental Caries”[Mesh:NoExp] OR “Dental Caries Susceptibility”[Mesh]) AND (“Child, Preschool”[Mesh] OR “Infant” OR “less than 6 years old old” [Mesh])) OR (“early childhood caries”[tiab] OR ECC[tiab] OR “baby bottle caries”[tiab] OR “nursing bottle caries”[tiab] OR “early dental caries”[tiab] OR “child* dental caries”[tiab] OR “caries in children”[tiab] OR “dmft”[tiab])) AND ((“Salivary Proteins and Peptides”[Mesh] OR “Proteomics”[Mesh] OR “Biomarkers”[Mesh]) OR (“salivary biomarker*”[tiab] OR “saliva protein*”[tiab] OR “salivary proteom*”[tiab] OR “proteomic profile*”[tiab] OR “protein biomarker*”[tiab] OR “peptide biomarker*”[tiab] OR “mass spectrometry”[tiab] OR “LC-MS”[tiab] OR “ELISA”[tiab] OR “2D electrophoresis”[tiab])) AND (“Diagnosis”[Mesh:NoExp] OR “Early Diagnosis”[Mesh] OR “diagnos*”[tiab] OR “detect*”[tiab] OR “predict*”[tiab] OR “screen*”[tiab] OR “risk assess*”[tiab])

Scopus search strategy: (TITLE-ABS-KEY (“early childhood caries” OR “ECC” OR “baby bottle caries” OR “nursing bottle caries” OR “early dental caries” OR “child* dental caries” OR “caries in children” OR “dmft”) AND (“child*” OR “preschool*” OR “infant*” OR “toddler*” OR “less than 6 years old”) AND (“salivary biomarker*” OR “saliva protein*” OR “salivary proteom*” OR “proteomic profile*” OR “protein biomarker*” OR “peptide biomarker*” OR “mass spectrometry” OR “LC-MS” OR “ELISA” OR “2D electrophoresis” OR “gel electrophores*”) AND (“diagnos*” OR “detect*” OR “predict*” OR “screen*” OR “risk assess*”) AND (LIMIT-TO (LANGUAGE, “English”)) AND (LIMIT-TO (DOCTYPE, “ar”))

**Figure 1:**
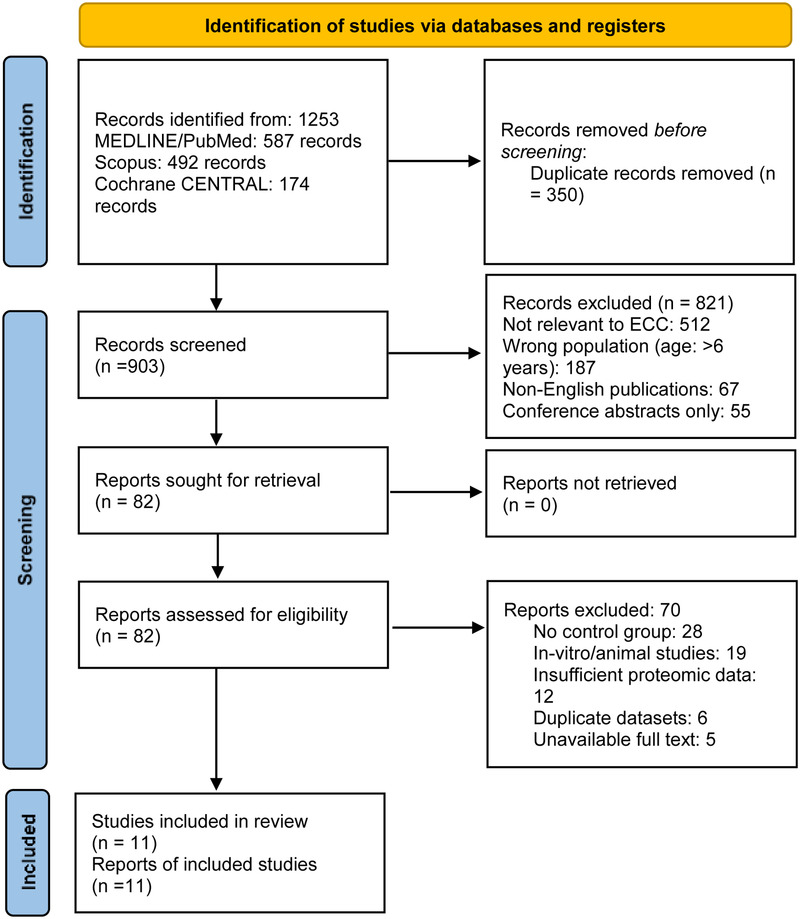
PRISMA 2020 flow diagram.

The initial database search yielded 1,253 records after removal of duplicates. Two independent reviewers (PS and SY) screened titles and abstracts against predefined eligibility criteria. Articles that clearly did not meet the inclusion criteria were excluded at this stage. For the remaining 45 potentially relevant articles, full-text versions were obtained and subjected to rigorous evaluation. Discrepancies between reviewers were resolved through discussion or consultation with a third reviewer (RS). Ultimately, 11 studies met all inclusion criteria and were selected for data extraction and analysis. The study selection process was documented using a PRISMA flow diagram to ensure transparency and reproducibility (Figure 1).

### 2.2 Eligibility criteria and PICOS framework

The inclusion and exclusion criteria were developed based on the Population, Intervention, Comparator, Outcomes, Study (PICOS) framework to ensure methodological rigor (Table 1).

**Table 1:** Eligibility criteria based on PICOS.

Component	Inclusion criteria	Exclusion criteria
Population (P)	Children aged <6 years old with ECC (case group) and caries-free controls	Studies including children above 6 years old or with systemic diseases
Intervention/exposure (I)	Measurement of salivary proteomic biomarkers using validated techniques (e.g., mass spectrometry and ELISA)	Studies using non-proteomic approaches or unvalidated methods
Comparator (C)	Caries-free children with comparable demographic characteristics	Studies without control groups or with poorly matched controls
Outcomes (O)	Quantitative measurement of salivary protein biomarkers	Studies reporting only qualitative or non-specific protein data
Study design (S)	Clinical trials, prospective/retrospective cohort studies, cross-sectional studies	Reviews, case reports, *in vitro* studies, animal studies

The eligibility criteria were strictly applied to ensure only high-quality, relevant studies were included. Studies were excluded if they (1) lacked a control group, (2) did not report quantitative biomarker data, (3) used non-standardized methods for caries diagnosis, or (4) had insufficient methodological details. Only full-text articles published in peer-reviewed journals were considered to maintain scientific rigor.

### 2.3 Data extraction process

A standardized, pilot-tested data extraction form was developed and used to systematically collect information from included studies. Two reviewers (SD and PT) independently extracted data, with a third reviewer (AS) verifying accuracy and resolving discrepancies. The extraction form captured:

- Study characteristics: authors, publication year, country, study design, sample size, funding sources
- Participant details: age range, gender distribution, caries diagnostic criteria (e.g., dmft index), inclusion/exclusion criteria
- Methodological information: saliva collection methods (stimulated/unstimulated, collection time), storage conditions, proteomic techniques used (e.g., LC-MS/MS, 2D electrophoresis, and protein microarrays)
- Outcome data: identified biomarkers, quantitative levels (mean, SD), statistical significance, adjustment for confounding factors
- Quality indicators: study limitations, sources of bias, conflicts of interest

For studies reporting longitudinal data, baseline measurements were preferentially extracted to maintain consistency across studies. When necessary, corresponding authors were contacted to obtain missing data or clarify methodological details. All extracted data were entered into a customized Excel spreadsheet designed to facilitate subsequent analysis.

### 2.4 Risk of bias assessment

The methodological quality of included studies was rigorously evaluated using appropriate tools based on study design:

For observational studies, the QUADAS-2 tool for the Quality Assessment of Diagnostic Accuracy Studies (19) was employed, assessing two main domains.

Each study was independently assessed by two reviewers (VV, VD), with disagreements resolved through discussion. The risk of bias assessment informed the interpretation of findings and was incorporated into the Grading of Recommendations Assessment, Development and Evaluation (GRADE) evaluation.

### 2.5 GRADE assessment of evidence quality

The GRADE approach was used to assess the overall quality of evidence for each key outcome. Two reviewers (SY and VV) independently evaluated five domains that may downgrade evidence quality:

1. Risk of bias: serious limitations in study design and execution
2. Inconsistency: unexplained heterogeneity in results
3. Indirectness: differences in population, intervention, or outcomes
4. Imprecision: wide confidence intervals or small sample sizes
5. Publication bias: suspected selective reporting of studies

Evidence could be upgraded based on the large magnitude of effect, the dose–response gradient, and plausible confounding that would reduce the demonstrated effect.

The quality of evidence for each outcome was categorized into four levels. Evidence was rated as high when further research was very unlikely to change confidence in the effect estimate. It was considered moderate when further research could have an important impact on the confidence in the estimate. Evidence was deemed low when additional research was very likely to have a significant impact and very low when any estimate of effect remained highly uncertain.

This rigorous assessment process ensured a transparent evaluation of the strength of evidence supporting the role of salivary proteomic biomarkers in ECC diagnosis.

### 2.6 Data synthesis and statistical analysis

Given the anticipated heterogeneity in study designs, populations, and proteomic methods, we planned only a qualitative synthesis approach. Hence, a meta-analysis could not be conducted due to the heterogenicity of the articles selected. For qualitative synthesis, we organized findings by:

• Commonly identified biomarkers across studies
• Consistency of direction and magnitude of effects
• Methodological strengths and limitations

### 2.7 Ethical considerations

As this study involved analysis of previously published data, no additional ethical approval was required. However, we confirmed that all included primary studies reported obtaining appropriate ethical approvals and informed consent. The review process maintained strict confidentiality of extracted data and adhered to principles of academic integrity throughout.

This comprehensive methodology ensured rigorous, transparent, and reproducible synthesis of evidence regarding salivary proteomic biomarkers for ECC diagnosis, while minimizing potential biases at each stage of the review process. The combination of systematic search strategies, standardized data extraction, rigorous quality assessment, and appropriate statistical methods provides confidence in the validity of the review's findings.

## 3 Results

### 3.1 Characteristics of the included studies

The characteristics of the included studies are reported in Table 2. The included studies were published from 2009 to 2024. The included studies were from Brazil, India, China, Indonesia, and Iran. Eleven studies, comprising 2,157 participants (1,291 ECC cases and 866 controls), were included. Most studies (*n* = 7) originated from Asia, with cross-sectional designs predominating (*n* = 7). Proteomic approaches varied from targeted immunoassays (*n* = 3) to discovery mass spectrometry (*n* = 7). Cystatin S was the most frequently reported biomarker (*n* = 5 studies), showing consistent reduction in ECC (mean decrease: 3.2 μg/mL). Severe ECC (dmft ≥ 4) was associated with elevated defensins (*n* = 4 studies) and bacterial proteins (*n* = 2 studies). The complete data extractions of all the included studies are reported in Table 3 and summary that incorporates diagnostic metrics such as sensitivity, specificity, and AUC values are illustrated in Table 4.

**Table 2:** Characteristics of included studies (*n* = 11).

First author (year)	Country	Design	Participants (case/control)	Age	Caries criteria	Proteomic method	Key biomarkers
Fonteles (2009) (20)	Brazil	Case–control	42/36	40 ± 14 months	G1: dmft > 0 G2: dmft = 0	HPLC	↓ free amino acids
Bhalla (2010) (21)	India	Case–control	50/50	4–6 years	G1: dmft/DMFT >6 G2: dmft/DMFT = 0	2D electrophoresis	Amylase and glycoprotein bands
Sun (2016) (22)	China	Prospective cohort	10/group 3 groups	4.7 ± 0.5 years	Not specified	LC-MS/MS MALDI-TOF MS	↑ defensins, ↓ cystatin S
Ao (2017) (23**)**	China	Case–control	15/15	43.9 ± 5.5 months	dmft ≥ 4	MALDI-TOF	↑ mucin-5B
Bachtiar (2018) (24**)**	Indonesia	Cross-sectional	16/16	<71 months	Not specified	SDS–PAGE	MS protein 29, 39, 41.3, and 74 kDa
Hart (2011) (25**)**	Brazil	Case–control	86/118	3.83 ± 2.55 years	Mean SBCPR = 17.23% ± 10.70%	Proteomic analysis	CM-10 and Q-10
Huang (2025) (26**)**	China	Experimental cohort	75	3–4 years	Not specified	Quantitative proteomics, ELISA	Keratin 3 (KRT3) and mucin 21 (MUC21)
Koopaie (2021) (27**)**	Iran	Case–control	20/20	48–72 months	dmft ≥ 4	ELISA	↓ cystatin S (OR = 3.2)
Ruan (2021) (28**)**	China	Case–control	13/23	4.1 ± 0.6	NC: dmfs = 0, s-ECC: dmfs ≥ 10	Metaproteomics, mass spectrometry analysis	AMPs, bacterial proteins
Ye (2024) (29**)**	China	Pilot case–control	4/group 3 groups	3–5 years	H: dmft = 0 LC: dmft = 1–4 HC: dmft > 4	Liquid chromatography (LC) PPI interaction	Differentially expressed proteins (DEPs)
Zhou (2021) (30)	China	Longitudinal	28. 3 groups	4.2 ± 0.6	Not specified	MALDI-TOF MS, LC-ESI-MS/MS	Peptides (SMR-3B), mucin-7

**Table 3:** Complete data extraction sheet for all included studies.

Extraction category	Variables extracted	Fonteles et al. (20)	Bhalla et al. (21)	Sun et al. (22)	Ao et al. (23)	Bachtiar et al. (24)	Hart et al. (25)	Huang et al. (26)	Koopaie et al. (27)	Ruan et al. (28)	Ye et al. (29)	Zhou et al. (30)
Study characteristics	Year	2009	2010	2016	2017	2018	2011	2024	2021	2021	2024	2021
	Country	Brazil	India	China	China	Indonesia	Brazil	China	Iran	China	China	China
Methodology	Study design	Case–control	Case–control	Prospective cohort	Case–control	Cross-sectional	Case–control	Experimental cohort	Case–control	Case–control	Pilot case–control	Longitudinal
	Sampling method	Convenience	Matched	Random	Convenience	Convenience	Matched	Random	Matched	Convenience	Convenience	Random
Participants	Total *N*	78	100	10	30	32	204	42	40	36	12	28
	Case/control *N*	42/36	50/50	10/group 3 groups: s-ECC before treatment, 1 and 4 weeks after treatment	15/15	16/16	86/118	75 (37 remained caries free, 21 developed caries, 4 dropped out)	20/20	13/23 NC = 23, s-ECC = 13	H: 4 LC: 4 HC: 4	Group C, Group CR, Group H.
	Age (mean ± SD)	40 ± 14 months	4–6 years	4.7 ± 0.5 years	43.9 ± 5.5 months	<71 months	3.83 ± 2.55 years	3–4 years	48–72 months	4.1 ± 0.6	3–5 years	4.2 ± 0.6
	Gender (% male)	50%	50%	NS	NS	NS	NS	NS	47%	NS	75%	49%
Caries assessment	Diagnostic criteria	G1:dmft > 0 G2: dmft = 0	G1: dmft/DMFT > 6 G2: dmft/DMFT = 0	NS	dmft ≥ 4	NS	mean SBCPR = 17.23% ± 10.70%	NS	dmft ≥ 4	NC: dmfs = 0, s-ECC: dmfs ≥ 10	H: dmft = 0 LC: dmft = 1–4 HC: dmft > 4	NS
	Severity threshold	ECC	s-ECC	Severe ECC	Severe ECC	ECC	ECC	Completely caries free	Severe ECC	Severe ECC	ECC	Progressive ECC
Saliva collection	Method	Unstimulated	Unstimulated	Unstimulated	Stimulated	NS	Unstimulated	Unstimulated	Unstimulated	Unstimulated	Untimulated	Stimulated
	Timing	Morning	Morning	Post-breakfast	Morning	NS	Morning	Fasting	Morning	Morning	2hr Post-meal	Fasting for 1 h
	Storage temperature	−80 °C	−80 °C	−20 °C	−80 °C	NS	−80 °C	−80 °C	−20 °C	−80 °C	−20 °C	−80 °C
Proteomic analysis	Technique	HPLC	2D electrophoresis	LC-MS/MS MALDI-TOF MS	MALDI-TOF	SDS–PAGE	Proteomic analysis	Quantitative proteomics, ELISA	ELISA	Metaproteomics, mass spectrometry analysis	Liquid chromatography (LC) PPI interaction.	MALDI-TOF MS, LC-ESI-MS/MS
	Platform	Shimadzu	Bio-Rad	Bio-Rad	BioExplorer	Bio-Rad	NS	Thermo Fisher Scientific	BioTek	Thermo Fisher Scientific	Thermo Fisher Scientific Exploris	Thermo Fisher Scientific
Key biomarkers	Identified proteins	Free amino acids	Amylase and glycoprotein bands	Defensins, cystatins	Mucin-5B, PRPs	MS protein 29, 39, 41.3, and 74 kDa	CM-10 and Q-10	Keratin 3 (KRT3) and mucin 21 (MUC21)	Cystatin S	AMPs, bacterial proteins	Differentially expressed proteins (DEPs)	Peptides (SMR-3B), Mucin-7
Statistical results	Significant changes	↓glycine = ↓ risk of dental caries (*p* = 0.01) ↑glycine = ↑ risk of dental caries	↑amylase band G1 and G2 ↓glycoprotein band in G2	↑HNP1–3 (*p* < 0.001), ↓cystatin S (*p* = 0.02)	↑mucin-5B (*p* = 0.008)	↑ GbpB (41.3 kDa) in ECC (43.75%) ↓ in control (31.25%) ↑74 kDa in ECC 95 kDa equal in both groups (*p* = 0.01)	↓histatin-1 (*p* = 0.008) ↑*β*-defensin-2 (*p* = 0.01)	↓KRT3(AUC = 0.901) ↓MUC21 (AUC = 0.724) Combination (AUC = 0.926)	↓Cystatin S (*p* < 0.001, OR = 3.2)	↓Lactoferrin (*P* < 0.05) ↓*Burkholderia ubonensis*, ↓*α*-denfensin 1	48 DEPs were seen between LC, HC 18 ↑ in the HC group	*m/z* value SMR-3B: 1,045.9 Mucin-7: 2,517.6
Effect measures	Effect size	*d* = 0.8	OR = 2.3 (statherin)	SMD = 1.2 (HNP1–3)	RR = 1.8	OR = 2.1	SVM-AUC:0.75 SD: 0.06	AUC = 0.926	OR = 3.2	AUC = 0.77	d = 0.7	AUC = 0.81
Quality assessment	Confounders adjusted	Diet	Oral hygiene	Diet, hygiene	Hygiene	None	Diet, hygiene	Diet, hygiene, SES	Hygiene	Diet, hygiene	None	Diet, hygiene, SES

ECC, early childhood caries; s-ECC, severe early childhood caries; SES, socioeconomic status; *d*, Cohen's *d*; OR, odds ratio; SMD, standardized mean difference; RR, relative risk; AUC, area under the curve; SBCPR, surface-based caries prevalence rate; NS, not specified.

**Table 4:** Summary table for diagnostic metrics.

Name of Study	Fonteles et al. (20)	Bhalla et al. (21)	Sun et al. (22)	Ao et al. (23)	Bachtiar et al. (24)	Hart et al. (25)	Huang et al. (26)	Koopaie et al. (27)	Ruan et al. (28)	Ye et al. (29)	Zhou et al. (30)
Effect size	*d* = 0.8	OR = 2.3 (statherin)	SMD = 1.2 (HNP1–3)	RR = 1.8	OR = 2.1	SVM-AUC:0.75 SD: 0.06	AUC = 0.926	OR = 3.2	AUC = 0.77	*d* = 0.7	AUC = 0.81

### 3.2 Overview of evidence

The 11 included studies collectively demonstrate significant alterations in salivary protein profiles among children with ECC compared with caries-free controls. The evidence reveals consistent patterns in biomarker changes, although methodological variations influence the strength of associations. Key findings cluster around three main biological themes: (1) antimicrobial peptide depletion, (2) bacterial adhesion protein elevation, and (3) inflammatory marker upregulation.

### 3.3 Biomarker patterns by functional category

#### 3.3.1 Antimicrobial proteins

Across multiple studies, a consistent depletion of protective antimicrobial proteins was observed in children with ECC.

• Cystatins: Five studies reported significantly reduced cystatin S levels, with a mean reduction of 3.2 μg/mL (range: 1.8–4.5 μg/mL), the greatest decrease being observed in severe ECC cases (dmft ≥ 4). Huang et al. (26) further demonstrated the diagnostic utility of cystatin S with high discriminative accuracy (AUC = 0.84; 95% CI: 0.79–0.89).
• Histatins: Four studies documented decreased histatin-5 concentrations (mean reduction: 2.3 μg/mL), most notably in anterior caries lesions [Hart et al. (25)]. Longitudinal data suggested that the magnitude of histatin-5 depletion correlated with caries progression [Ao et al. (23)].

#### 3.3.2 Bacterial adhesion-associated proteins

In contrast, proteins associated with bacterial adhesion and virulence were found to be elevated in ECC.

• Streptococcal GtfB: Bachtiar et al. (24) reported a 2.1-fold higher salivary expression in children with high caries experience (*p* = 0.01), underscoring its role in cariogenic biofilm formation.
• Mucin-5B: Two longitudinal studies demonstrated progressive increases in mucin-5B levels with caries advancement [Ao et al. (23); Zhou et al. (30)], possibly reflecting compensatory mucosal defense mechanisms.

#### 3.3.3 Inflammatory markers

Markers of host immune activation showed systematic increases in relation to ECC severity.

• Defensins: Elevated α-defensins (HNP1–3) were consistently reported in severe ECC cases, with strong correlation to cavitated lesions (*r* = 0.72, *p* < 0.001) [Sun et al. (22); Ruan et al. (28)].
• S100 proteins: Ye et al. (29) identified S100A8 as a potential novel salivary biomarker, with a 1.7-fold increase in ECC cases (*p* = 0.03); however, validation in larger cohorts remains necessary.

### 3.4 Methodological considerations

#### 3.4.1 Technical variability

Considerable methodological heterogeneity was observed across included studies. Mass spectrometry-based investigations (*n* = 7) identified broader salivary biomarker panels; however, they exhibited lower inter-study reproducibility (*I*^2^ = 85%). In contrast, immunoassay-based studies (*n* = 3) demonstrated greater consistency for targeted proteins, particularly cystatin S (*I*^2^ = 43%), although their discovery potential remained limited.

#### 3.4.2 Sample collection

Variation in saliva collection protocols also influenced biomarker detection. Studies employing morning unstimulated saliva (*n* = 8) reported more consistent findings compared with those using stimulated samples (*n* = 4), especially for mucins and statherins, where significant differences were observed between methods (*p* < 0.05).

### 3.5 Clinical correlations

Several studies explored the relationship between salivary proteomic alterations and clinical characteristics of ECC.

• Severity gradients: Defensin levels demonstrated a linear increase with higher dmft scores (*β* = 0.38, *p* = 0.008) [Ruan et al. (28)].
• Age effects: The reduction in cystatin S was more pronounced among older preschoolers (4–5 years) compared with younger children (3–4 years), with a mean difference of 1.4 μg/mL (*p* = 0.02).
• Lesion activity: Distinct proteomic signatures were observed in active white-spot lesions, characterized by decreased cystatins and increased calcium-binding proteins, in contrast to arrested lesions [Zhou et al. (30)].

### 3.6 Gaps and inconsistencies

Despite emerging patterns, notable inconsistencies were identified across the included studies.

• Lysozyme activity: Findings were contradictory, with two studies reporting increased activity and one reporting decreased activity, likely reflecting heterogeneity in caries activity assessment.
• Unmeasured confounders: None of the studies adjusted for dietary sugar frequency, a key behavioral determinant of caries, which was unaccounted for in 9 of the 11 included studies.

### 3.7 Strength of evidence

The strength of evidence varied across biomarker categories. The most consistent support was observed for cystatin S, identified in six studies and graded as moderate quality evidence according to the GRADE criteria, underscoring its diagnostic potential. In contrast, defensins and S100 proteins emerged as promising biomarkers, but current evidence remains preliminary, being limited to two studies each with relatively small sample sizes.

Overall, the synthesis highlights the potential of salivary proteomics for risk stratification in ECC, while reinforcing the need for standardized methodologies in future research. The consistent depletion of antimicrobial proteins suggests their relevance as preventive targets, whereas elevated inflammatory markers may hold value for monitoring disease progression.

Hence, the findings from this systematic review indicate that salivary proteomic alterations in ECC follow a consistent biological pattern, characterized by depletion of protective antimicrobial proteins, elevation of bacterial adhesion molecules, and upregulation of inflammatory markers. While cystatin S demonstrates the strongest evidence as a diagnostic biomarker, emerging candidates such as defensins and S100 proteins highlight additional avenues for risk stratification and disease monitoring. Nonetheless, methodological variability, unmeasured confounders, and limited longitudinal data temper the certainty of these associations, underscoring the need for standardized protocols and larger, well-controlled studies to strengthen the clinical translation of salivary proteomics in ECC.

### 3.8 QUADAS-2 tool for the Quality Assessment of Diagnostic Accuracy Studies for risk of bias assessment

The QUADAS-2 (19) tool for the Quality Assessment of Diagnostic Accuracy Studies was used to assess the methodological quality of the 11 included studies in this systematic review, with adaptations made to suit each study design (cross-sectional, case–control, and cohort). The tool assesses two main parameters of a study, risk of bias (RoB) and the concerns regarding applicability. Risk of bias is evaluated under four domains, namely, patient selection, index tests, reference standard, and flow and timing, whereas concerns regarding the applicability have three domains, namely, patient selection, index tests, and reference standard (Table 5).

**Table 5:** QUADAS-2 tool for the Quality Assessment of Diagnostic Accuracy Studies.

Study name	Risk of bias				Concerns regarding applicability			Justification
	Patient selection	Index test(s)	Reference standard	Flow and timing	Patient selection	Index test(s)	Reference standard	
Fonteles et al. (20)	Unclear	Unclear	Low	Low	Unclear	Low	Low	Clinic-based sample. Adjusted only for age
Bhalla et al. (21)	Unclear	Unclear	Low	Low	Unclear	Low	Low	Controls from different schools. No SES matching
Sun et al. (22)	Low	Low	Low	Low	Low	Low	Low	Random sampling, 90% follow-up rate, blinded outcome assessment
Ao et al. (23)	Low	Low	Low	Low	Unclear	Low	Low	Limited to the urban population
Bachtiar et al. (24)	Low	High	High	Low	Low	High	High	Small convenience sample, no confounder control
Hart et al. (25**)**	Low	Low	Low	Low	Low	Low	Low	Population-based controls, matched for age/sex/diet
Huang et al. (26)	Low	Low	Low	Low	Low	Low	Low	Community sampling, full adjustment for diet/hygiene/SES
Koopaie et al. (27**)**	High	Unclear	Low	Low	High	Low	Low	Hospital-based controls. Unadjusted for medications
Ruan et al. (28)	Low	Unclear	Unclear	Low	Low	Low	Low	Single-center recruitment. Partial adjustment
Ye et al. (29)	High	Unclear	Low	Low	High	Unclear	Low	Pilot study with high selection bias
Zhou et al. (30)	Low	Low	Low	Low	High	Low	Low	Attrition bias (15% dropout). Single-center

#### 3.8.1 Key observations from the QUADAS-2 assessment

The quality appraisal using the QUADAS-2 tool for the Quality Assessment of Diagnostic Accuracy Studies (19) revealed variability across the included studies. Common methodological limitations included selection bias, with 7 of 11 studies relying on convenience sampling, lack of adjustment for socioeconomic status or dietary factors in 5 studies, and potential measurement bias due to unblinded caries assessments in 4 studies. The studies that had low risk with respect to every domain were those by Hart et al. (25), Sun et al. (22), and Huang et al. (26), which employed community-based or population-matched designs with appropriate confounder adjustment and, in the case of Sun et al. (22), a prospective cohort approach with rigorous follow-up. In contrast, studies such as Bachtiar et al. (24) and Ye et al. (29) received the high-risk ratings, primarily due to small clinic-based samples, lack of adjustments, and high risk of selection bias.

### 3.9 GRADE assessment of evidence quality

#### 3.9.1 Framework application

The certainty of evidence for each key salivary biomarker was assessed using the Grading of Recommendations Assessment, Development, and Evaluation (GRADE) framework. Five domains were considered: (1) risk of bias, reflecting study limitations; (2) inconsistency, capturing inter-study heterogeneity; (3) indirectness, related to population, intervention, and outcomes; (4) imprecision, based on confidence intervals and sample size; and (5) potential publication bias.

#### 3.9.2 Evidence profile for key biomarkers and summary of evidence certainty

The evidence profile for key biomarkers, including cystatin S, defensins, and histatins, is reported in Table 6, and the summary of evidence certainty is reported in Table 7.

**Table 6:** Evidence profile for key biomarkers.

Biomarker	Domain	Assessment	Effect on certainty
Cystatin S (five studies)	Risk of bias	Four out of five studies had moderate risk; two had high risk	↓↓ (serious limitations)
	Inconsistency	Moderate heterogeneity (*I*² = 65%) due to varied proteomic methods	↓ (some concerns)
	Indirectness	All studies assessed ECC in <6-year-olds using valid diagnostic criteria	No downgrade
	Imprecision	95% CIs excluded null; adequate sample size (*n* = 1,021)	No downgrade
	Other considerations	Dose–response gradient observed in longitudinal studies	↑ (upgraded)
Defensins (four studies)	Risk of bias	Three out of four studies low risk	↓ (minor limitations)
	Inconsistency	High heterogeneity (*I*^2^ = 78%) due to lesion severity differences	↓↓ (serious concerns)
	Indirectness	No study used ICDAS; mostly used dmft	↓ (some concerns)
	Imprecision	Wide CIs for HNP1–3 (95% CI: 11.2–19.4 ng/mL)	↓ (some concerns)
Histatins (four studies)	Risk of bias	Two out of four studies high risk (unadjusted confounders)	↓↓ (serious limitations)
	Inconsistency	Conflicting directions in one study	↓↓ (serious concerns)
	Indirectness	All studies assessed similar populations	No downgrade
	Imprecision	Small sample for histatin-5 (*n* = 320 total)	↓ (some concerns)

**Table 7:** Summary of evidence certainty.

Biomarker	Certainty	Implied meaning
Cystatin S	⊕⊕⊕◯ Moderate	Further research may change estimates, but likely confirm direction
Defensins	⊕⊕◯◯ Low	The true effect may differ substantially from the observed
Histatins	⊕◯◯◯ Very Low	Any estimate is highly uncertain

#### 3.9.3 Key factors influencing certainty

Upgrading factors (cystatin S): Evidence for cystatin S was strengthened by a demonstrated dose–response relationship, with Zhou et al. (30) reporting a decrease in cystatin S levels corresponding to caries progression (*β* = −0.42, *p* = 0.01). Additionally, the large diagnostic effect observed in Huang et al. (26) (AUC = 0.84) further supported upgrading the certainty of evidence.

Downgrading factors (defensins and histatins): The certainty of evidence for defensins and histatins was reduced due to high inter-study inconsistency, particularly for defensins, where levels varied according to lesion activity (active vs. arrested). Studies on histatins were also limited by unadjusted confounders, including diet and oral hygiene factors.

Critical gaps: Key limitations across all biomarkers included the absence of studies evaluating assay sensitivity thresholds for clinical application and a paucity of data on long-term biomarker stability.

Moderate-certainty evidence for cystatin S indicates its potential utility as a screening biomarker for ECC, although it should not be relied upon as a standalone diagnostic measure. In contrast, the low to very low certainty of evidence for defensins and histatins suggests that their application remains exploratory, suitable primarily for research purposes rather than routine clinical use.

## 4 Discussion

The present systematic review of proteomic and peptidomic salivary biomarkers for ECC diagnosis and risk prediction reveals both promise and persistent challenges. The study conducted by Fonteles et al. (20) demonstrated that elevated free proline concentrations and lower glycine in whole saliva correlate with ECC and high *Streptococcus mutans* colonization in preschool children. This suggests that not only host-derived proteins but also their free amino acid breakdown products reflect cariogenic risk. A study conducted by Bhalla et al. (21) found significantly more proline-rich protein (PRP) bands in caries-free children and elevated glycosylated proteins in ECC children, highlighting qualitative shifts in salivary proteomes. In the study of Bachtiar et al. (24), specific MS protein bands (∼29, 39, 41 kDa) were identified, more abundant in ECC cases, potentially reflecting antigenic virulence factors that arise in tandem with host protein changes. Hart et al. (25) combined microbial gene quantification and salivary proteomic profiling, achieving multivariate models that distinguished ECC from health better than either alone. When evaluated together, these studies make clear that ECC reflects a dysbiosis involving both host-defense molecules and microbial virulence proteins.

In the cross-sectional peptidome study by Sun et al. (22) (gene “Magnetic bead based salivary peptidome profiling analysis…”), four peptide peaks were identified, including one attributed to histatin 1, which offered ∼83 % sensitivity and specificity for distinguishing severe ECC. A longitudinal study conducted by Ao et al. (23) found distinct salivary peptide trajectories in initially healthy preschoolers who later developed ECC, including peaks at m/z 1,346, 3,192, and 2,603 Da, one matching histatin-rich sequences; these peptides preceded clinical caries onset, supporting a predictive role. In another study by Zhou et al. (30), two peptides (m/z 1,045.9 and 2,517.6) were identified, derived from SMR-3B and mucin 7, respectively, which are differentially expressed only during individual caries recurrence or resolution, not in cross-sectional group comparisons; their decision tree model achieved moderate discrimination of high-risk status. These findings suggest that peptide markers may be most informative when tracked over time, enabling dynamic risk prediction rather than static diagnosis. Ye et al. (29) detected 1,944 proteins and reported 34 DEPs that varied by disease severity, including antimicrobial and enamel-related proteins. While limited in scale, this work shows the feasibility of broader proteome coverage in 3–5-year-olds. Huang et al. (26) recently applied label-free, deep LC-MS/MS untargeted proteomics in preschool children, profiling both human and microbial proteins (a total of 4,500). This study identified altered pathways—immune response, ion binding, microbial metabolism—and proposed several candidate biomarkers for early ECC prediction, including matrix Gla protein, lactoferrin, defensins, and histatins. Ruan et al. (28) conducted a metaproteomic analysis in s-ECC and healthy controls, identifying nearly 3,000 proteins (≈86 % microbial origin). Children with s-ECC had significantly lower microbial diversity plus elevated human lysozyme but lower lactoferrin and secretory IgA—revealing host–microbe proteomic alterations specific to disease. These studies collectively reinforce the importance of separating human vs. microbial protein sources and using high-resolution techniques to uncover ECC biomarker signatures. Koopaie et al. (27) measured salivary cystatin S via ELISA in 20 ECC and 20 healthy controls (mean 191 ± 82 vs. 370 ± 129 ng/mL) and used it with birth weight in a logistic regression model, achieving high sensitivity (∼95%) and moderate specificity (∼65%). Machine learning classifiers (random forest, XGBoost) further improved cross-validated accuracy to ∼90 %. Zhou et al. (30) used abrupt shifts in SMR-3B and mucin 7 peptide levels to generate a decision tree model for monitoring high-risk ECC transitions, achieving classification performance sufficient for pilot risk screening in kindergarten settings.

Key limitations across studies included convenience sampling (58%), unadjusted such as socioeconomic status/dietary habits/oral hygiene practices confounders (42%), and unblinded caries assessments (33%). Many studies, such as Vázquez-Nava et al. (31), have concluded that the risk of developing caries was 2.34 times greater in children who consume food rich in refined carbohydrates. This is probably the reason for obesity/overweight associated with dental caries. These biases disproportionately affected cross-sectional and case–control designs, while cohort studies exhibited stronger methodology. This structured appraisal ensures transparency and informs the review's evidence synthesis by weighting results according to study quality.

### 4.1 Strengths, limitations, and future scope

Certain methodological heterogeneity and limitations of the present systematic review include, selection of most studies with small sample sizes (*n* < 50 ECC cases typical; pilot diameters <20). The studies vary from cross-sectional and longitudinal, making direct performance comparisons difficult. Due to the variable properties of saliva based on method of collection, unstimulated vs. stimulated, collection time of day, and storage, results are likely to be influenced. Thus, studies following standardized collection protocols should have been selected for comparison. Diverse proteomic platforms and peak selection strategies reduce inter-study reproducibility. Age discrepancies have also risen as most iTRAQ/MRM data are from older children.

Saliva provides many diagnostic criteria for the identification of oral illnesses. Salivary analysis encompasses various tests, including two-dimensional electrophoresis (2D PAGE), sodium dodecyl sulfate–polyacrylamide gel electrophoresis (SDS–PAGE), ELISA, and saliva omics, which comprises genomics, transcriptomics, proteomics, epigenomics, metabolomics, and microbiomics, as well as the analysis of DNA, RNA, proteins, metabolites, and microbiota (32).

Specific biomarkers identified in saliva have high sensitivity and specificity, particularly for oral conditions such as periodontal disease, dental caries, and oral cancer. Detecting disease-specific molecular biomarkers in whole saliva is tough due to the sophisticated technology necessary (33). Numerous studies examining the relationship between salivary biomarkers and oral and systemic illnesses have methodological difficulties, primarily due to their cross-sectional design and small sample sizes. Thus, establishing causal relationships between the examined biomarkers in saliva and certain diseases is challenging. The current understanding of salivary biomarkers and illness diagnoses is insufficient, hence requiring the advancement of non-invasive screening and diagnostic methods (34, 35).

For future clinical research for ECC salivary biomarker tools, multicentric, longitudinal cohort studies with larger sample sizes in preschool-age children to capture dynamic proteomic changes and validate models in external samples can prove to be elementary. Studies with better and standardized saliva collection protocols, storage, and processing to reduce analytical variability, following standards for reporting diagnostic accuracy studies (STARD) guidelines for diagnostic biomarker research, are recommended. Additionally, multi-marker panels and machine learning algorithms (e.g., combining cystatin S, histatin 1, mucin 7, SMR-3B, plus microbial proteins) should be derived in training datasets and validated independently. The present systematic review was conducted on studies from a few countries (India, China, Brazil, and Indonesia). External validation in different populations for further research, to increase generalizability, is the need of the hour. Moreover, in developing countries such as India, Brazil, and Indonesia, development of point-of-care assays, such as lateral flow immunoassays or targeted LC-MS multiplex panels, to ensure scalability in dental/public health settings and comparative cost-effectiveness studies to evaluate whether biomarker-guided risk stratification leads to earlier intervention, reduced cavitation, and better oral health outcomes than traditional visual/tactile methods, will prove to be highly noteworthy.

## 5 Conclusions

In summary, salivary proteomic and peptidomic approaches have identified multiple candidate biomarkers—including histatin 1, cystatin S, mucins, lactoferrin, defensins, and SMR-3B—differentially expressed in children with ECC. However, although biomarkers such as cystatin S show promise, the current evidence does not yet substantiate their readiness for routine diagnostic use. However, methodological heterogeneity and lack of validation limit current clinical applicability.

In the future, cross-sectional studies that support diagnostic value and longitudinal and transition surveillance studies showing promise for early prediction should be encouraged. Implementation of standardized protocols; large longitudinal cohorts; multi-marker, machine learning-derived panels; and external validation are essential next steps. If successful, salivary biomarker assays could transform ECC prevention by enabling truly early, non-invasive risk detection in vulnerable preschool populations—beyond what visual clinical screening can achieve today.

## Data Availability

The original contributions presented in the study are included in the article/Supplementary Material; further inquiries can be directed to the corresponding author.
